# Web-Based Interactive Training for Managers (Managing Minds at Work) to Promote Mental Health at Work: Pilot Feasibility Cluster Randomized Controlled Trial

**DOI:** 10.2196/76373

**Published:** 2025-09-02

**Authors:** Juliet Hassard, Holly Blake, Teixiera Mishael Dulal-Arthur, Alexandra Frost, Craig Bartle, Joanna Yarker, Fehmidah Munir, Ben Vaughan, Guy Daly, Caroline Meyer, Sean Russell, Louise Thomson

**Affiliations:** 1 Queen's Business School Queen's University Belfast Belfast United Kingdom; 2 School of Health Sciences, University of Nottingham Nottingham United Kingdom; 3 NIHR Nottingham Biomedical Research Centre Nottingham United Kingdom; 4 School of Medicine, University of Nottingham Nottingham United Kingdom; 5 Institute of Mental Health, Nottinghamshire Healthcare NHS Foundation Trust Nottingham United Kingdom; 6 Birkbeck, University of London London United Kingdom; 7 Affinity at Work London United Kingdom; 8 School of Sport, Exercise and Health Sciences, Loughborough University Loughborough United Kingdom; 9 Office of the Provost, British University in Egypt Cario Egypt; 10 Faculty of Health and Life Sciences, Coventry University Coventry United Kingdom; 11 Medical School, University of Warwick Coventry United Kingdom

**Keywords:** line managers, web-based training intervention, mental health at work, feasibility pilot study

## Abstract

**Background:**

Line managers play a key role in preventing poor mental health but often lack necessary skills and knowledge. Existing interventions typically focus on mental health awareness rather than practical skills. The evidence-based Managing Minds at Work (MMW) web-based training program was developed to address this gap by enhancing line managers’ confidence and competence in prevention.

**Objective:**

This study piloted the MMW intervention to assess its feasibility. Objectives included evaluating (1) uptake potential across small, medium, and large companies; (2) perceived suitability and effectiveness of the intervention; and (3) feasibility of outcome data collection methods.

**Methods:**

We conducted a 2-arm pilot cluster randomized controlled trial of a self-guided, web-based training intervention for line managers. Twenty-four organizations were randomly assigned to the MMW intervention or a 3-month waitlist. A total of 224 line managers completed baseline measures (intervention: n=141, 62.9%; control: n=83, 37.1%), along with 112 of their direct reports (intervention: n=74, 66.1%; control: n=38, 33.9%). Follow-up data were collected at 3 and 6 months. Semistructured interviews with line managers and stakeholders (n=20) explored experiences with the study and intervention, and qualitative data were analyzed thematically. Line managers also completed feedback forms after each of the 5 MMW modules.

**Results:**

The recruitment of organizations and line managers exceeded targets, and retention rates of line managers were good at 3 months (161/224, 71.9%) but not at the 6-month follow-up (55/224, 24.6%). Feedback on the intervention was very positive, indicating that line managers and organizational stakeholders found the intervention acceptable, usable, and useful. We observed significant improvements with moderate to large effect sizes for all trial outcomes for line managers in the intervention arm from baseline to the 3-month follow-up. Line managers completed a variety of questionnaires, which showed increased scores for confidence in creating a mentally healthy workplace (intervention group: mean change 3.8, SD 3.2; control group: mean change 0.6, SD 3.2), mental health knowledge (intervention group: mean change 1.9, SD 3.0; control group: mean change 0.2, SD 2.9), psychological well-being (intervention group: mean change 3.6, SD 8.3; control group: mean change −0.7, SD 7.7), and mental health literacy at work (intervention group: mean change 11.8, SD 8.9; control group: mean change 0.8, SD 6.2). Collecting data from direct reports in both study arms was challenging, with results inconclusive regarding observed changes in trial outcomes. Time constraints and workload were commonly cited barriers to completion of the intervention.

**Conclusions:**

This pilot feasibility trial provides strong evidence for the usability and acceptability of the MMW digital training and the research design. MMW shows potential to improve line managers’ confidence and competencies in promoting mental health. The study also identified key considerations for future large-scale implementation and evaluation.

**Trial Registration:**

ClinicalTrials.gov NCT05154019; https://clinicaltrials.gov/study/NCT05154019

## Introduction

### Background

It is estimated that 15% of the global working-age population have a mental health condition [1]. This has been exacerbated by the COVID-19 pandemic, which led to a rise in mental ill-health among working-age adults [2,3]. In the United Kingdom, approximately 7.9% of sickness absence was due to mental health conditions [4], and more than half of all working days (approximately 17 million) were lost due to depression, anxiety, and work-related stress in 2021 to 2022 [3]. The human and economic costs of poor mental health at work are profound, impacting both employers and society at large [5,6]. It is estimated that poor mental health across the UK workforce is costing employers £51 billion (US $68.4 billion) per annum [7]. Despite these costs, many employers lack awareness of their responsibility to support the mental health of their employees [2], with many lacking provisions or policies to promote employee psychological well-being [8].

Workplace mental health interventions are typically categorized as primary (taking action to eliminate the sources of stress or poor mental health in the workplace), secondary (detection and management of experienced stress and poor mental health by increasing awareness, knowledge, skills, and coping resources), or tertiary (minimizing the effects of poor mental health at work once they have occurred through treatment of symptoms). Best practice advocates for a holistic approach integrating all 3 levels of intervention, targeting both workplace- and worker-directed strategies [9,10]. In particular, primary prevention is crucial to maximize employee health and productivity [8,10] and is clearly emphasized in national (eg, from Canada [11], Australia [12], and the United Kingdom [13]) and international (eg, International Organization for Standardization standard 45003 [14], the International Labour Organization [15], and the World Health Organization [1]) standards on promoting mental health at work and preventing work-related stress. The need for and use of prevention-oriented approaches is strongly emphasized across these national and international standards, with reference to the central and ongoing role played by line managers throughout this process.

The role that line managers play in preventing work-related stress and promoting better mental health at work includes designing and managing work tasks, communicating with employees respectfully and clearly, fostering psychologically safe and supportive team environments, and encouraging open dialogue about mental well-being [16-21]. These responsibilities are central to a primary prevention approach, which targets work-related stressors before they result in harm. Drawing on the job demands–resources (JD-R) model [22], job demands refer to aspects of a job that require sustained physical or psychological effort (such as workload, time pressure, and emotional demands), whereas job resources refer to elements that help achieve work goals, reduce demands, or stimulate growth and development (such as role clarity, supervisory support, and autonomy).

**Figure 1:**
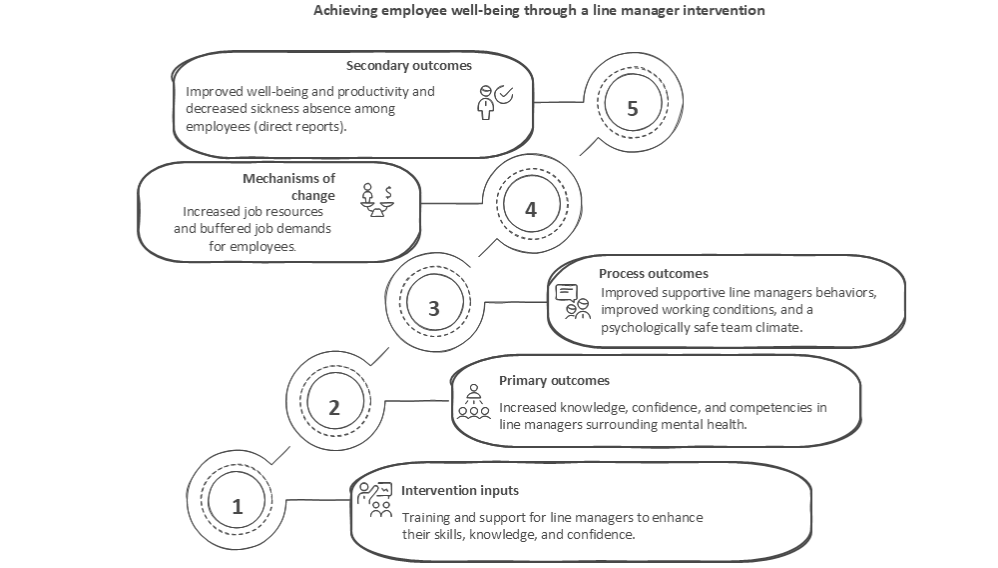
Conceptual framework linking the line manager (LM) intervention to mental health and well-being outcomes.

The intervention program theory, developed during the earlier Medical Research Council guided intervention development phase [23], outlines how the Managing Minds at Work (MMW) training is expected to enhance these job resources by improving line manager knowledge, confidence, and behavioral competencies (*primary outcomes*). These improvements are theorized to lead to more supportive, clear, and psychologically safe management practices (*process outcomes*), which in turn help buffer the impact of job demands and create more favorable psychosocial work environments. Ultimately, these mechanisms are hypothesized to improve employee well-being, perceptions of managerial competency, and productivity and reduce sickness absence behaviors (*secondary outcomes*). These pathways are visually represented in the conceptual model in Figure 1, which maps the theorized links between intervention components and outcomes at the individual and organizational levels.

Before the COVID-19 pandemic, approximately half of line managers had not been offered the necessary training to support them in these managerial duties and responsibilities [24,25]. However, recent figures indicate an upward trend of enterprises now offering mental health training for line managers as part of their well-being practices [26], with growing evidence of their impact on managers’ behaviors and well-being [27] and organizational outcomes [26,28-30]. This underscores the need for evidence-based line manager training interventions focused on developing their knowledge, confidence, and behavioral competencies to prevent work-related stress and promote mental health at work.

Recent evidence and systematic reviews have highlighted a lack of robust evaluations of managerial or leadership training to improve employee well-being [10,31-33]. There is a limited number of line manager–targeted intervention studies using trial methodology in the literature [32,34-40]. There are 2 notable gaps in this existing evidence base. First, only 4 of the line manager–targeted intervention studies include a broad prevention-oriented approach [32,36,39,40], only 3 of which are cluster randomized controlled trials (RCTs) [32,36,40]. Therefore, there is a need to develop and rigorously test a line manager training intervention underpinned by primary prevention. Second, most existing training interventions are delivered face-to-face rather than using a digital or hybrid (face-to-face and digital) delivery method except for those reported in 4 studies [32,36,39,40]. Digital interventions are increasingly being used within applied contexts (eg, workplaces [41]) due to their time- and resource-efficient nature [42-44]. However, little is known about their usability and acceptability to line manager recipients or indirect impact on those they manage (their direct reports), in particular for those digital interventions targeting knowledge and behavior change. The MMW digital training intervention seeks to address this evidence gap.

This study addresses key gaps in the existing literature by developing and testing a digital line manager training intervention explicitly grounded in primary prevention and informed theoretically by the JD-R model [22]. It contributes to the limited number of rigorously designed trials evaluating line manager training for mental health by using a cluster randomized controlled design, following the CONSORT-EHEALTH (Consolidated Standards of Reporting Trials of Electronic and Mobile Health Applications and Online Telehealth) guidelines [45], and ensuring transparency through trial preregistration (December 8, 2021) and protocol publication [46]. In doing so, this study strengthens methodological rigor in this area; supports standardization of intervention approaches; and generates insights into the feasibility, acceptability, and potential impact of digitally delivered line manager training. The findings will inform the design of a future definitive trial and help guide evidence-based workplace mental health strategies.

### Objectives

This study aimed to pilot and test the feasibility of a future definitive cluster RCT of the MMW digital training intervention [46].

The overarching objectives were to assess (1) the potential for uptake within small, medium, and large companies; (2) the perceived suitability and effectiveness of the line manager training; and (3) the feasibility of the data collection methods for primary and secondary outcome measures.

Table 1 provides an overview of the pilot feasibility trial objectives and the data sources used to investigate them.

**Table 1:** Overview of trial objectives and mapped data sources used to explore them.

Feasibility trial objectives		Data source and data collection method	Criterion
**The potential for uptake within small, medium, and large companies by identifying and monitoring several aspects (objective 1)**			
	Willingness of employers to register interest in participating in the trial and allow LMs^a^ to take part in the trial (objective 1.1)	End-of-study records used to examine the percentage of organizations converted from expressed interest to recruitment to the study	Minimum of 8 organizations (4 in each arm)
	Recruitment and retention rates of LMs in the trial (objective 1.2)	End-of-study records of percentage of eligible LMs who consented and percentage of consenting LMs who completed the 3- and 6-mo follow-ups	70% retention rate from baseline to follow-up assessments
	Recruitment and retention of LMs’ DRs^b^ (objective 1.3)	Study records of percentage of consenting DRs who completed the 3- and 6-mo follow-ups	50% retention rate from baseline to follow-up assessments
**The perceived suitability and effectiveness of the LM training by determining several aspects (objective 2)**			
	Acceptability, usability, and utility of the training among LMs and stakeholders within the participating organizations (objective 2.1)	Qualitative data from stakeholder postintervention interviews and qualitative feedback provided by LMs after each training module	—^c^
	Potential for improving LMs’ confidence (primary outcome) and knowledge, managerial behavioral competencies, and psychological well-being (secondary outcomes) to inform the planning of a larger trial (objective 2.2)	Web-based survey at baseline, completion of the intervention (intervention arm only), and 3 and 6 mo after baseline using psychometrically validated measures (see the Primary and Secondary Outcomes section)	Change in mean score over time in the intervention group as compared to the waitlist control arm
	Potential for improving employee outcomes (DRs’ well-being, absence, and productivity) and their perceptions of their LMs’ managerial competencies by assessing changes to inform the planning of a larger trial (objective 2.3)	Web-based survey at baseline, completion of the intervention (intervention arm only), and 3 and 6 months after baseline on DRs’ well-being, productivity, and perceptions of their LMs’ managerial behavioral competencies using psychometrically validated measures; sick absence information was collected through organizational records	Change in mean score over time in the DRs managed by LMs in the intervention group as compared to that among the DRs in the waitlist control arm
	Barriers to and facilitators of intervention implementation and effectiveness to inform the delivery of the intervention for the full trial (objective 2.4)	Qualitative postintervention data from interviews with stakeholders and qualitative feedback completed by LMs after each training module	—
The data collection methods for primary and secondary outcome measures to inform key parameters for a larger trial related to sample size and clustering (objective 3)		End-of-study qualitative data and researcher reflections to examine the willingness to allow access to organizational-level data on sickness absence	—

^a^LM: line manager.

^b^DR: direct report.

^c^No criterion specified, as thresholds were only set for quantitative outcomes to assess feasibility metrics such as recruitment and retention. For qualitative objectives, the focus was on generating rich, contextual insights into acceptability, usability, and implementation (objective 2), where predefined thresholds are not appropriate or meaningful.

## Methods

This study adhered to the CONSORT-EHEALTH guidelines [45] for pilot and feasibility trials and a focus on both implementation and outcome feasibility (Multimedia Appendix 1).

### Ethical Considerations

Ethics approval was granted by the University of Nottingham’s Faculty of Medicine and Health Sciences ethics committee (reference 299-0621, with an amendment approved on June 6, 2022). Written informed consent was obtained from all individual participants before participation, and organizational consent was secured from all participating companies before onboarding. Participant data were handled in accordance with data protection guidelines. All responses were anonymized, securely stored on encrypted drives common accessible only to the research team. No financial or material compensation was provided to participants or organizations for their involvement in the study.

### Design

MMW was evaluated using a multisite, 2-armed pilot and feasibility cluster RCT with an embedded qualitative study in organizations of different sizes and sectors in England [46]. The organizations (the clusters) were the specified units of randomization, and the line managers were the participants. Follow-up assessments from baseline were completed 3 and 6 months later. While pilot feasibility studies typically use shorter follow-up periods [23], we included both 3- and 6-month follow-up assessments to explore longer-term outcomes and acceptability. This extended follow-up was made possible due to additional project funding and was formally documented as a change in the trial registration and published study protocol [46].

Initially, a postintervention follow-up for the intervention arm was planned to be completed at the end of the intervention and estimated to be approximately 6 weeks after baseline. However, due to variation in the length of time taken to complete the intervention, for many participants, the 6-week postintervention follow-up was very close to the 3-month follow-up. Although we did not formally track completion of each module through the platform hosting MMW, optional end-of-module feedback forms allowed for an informal mechanism to monitor progress. These forms indicated some variation in the length of time taken to complete the intervention. To prevent participant burden and maintain a standardized 3-month follow-up across both arms, the 6-week postintervention follow-up was removed from the schedule. Those in the waitlist condition completed the 3-month follow-up assessment before gaining access to the intervention. Similarly, employees that the line managers managed (their direct reports) were assessed at baseline and 3 and 6 months later. Blinding was not applied in this study for pragmatic reasons (eg, the need to send reminder emails for intervention completion by the research team). Both the participants and organizations were aware of the condition to which they were assigned. The trial ended when the last participant had completed follow-up data collection.

### Recruitment

**Figure 2:**
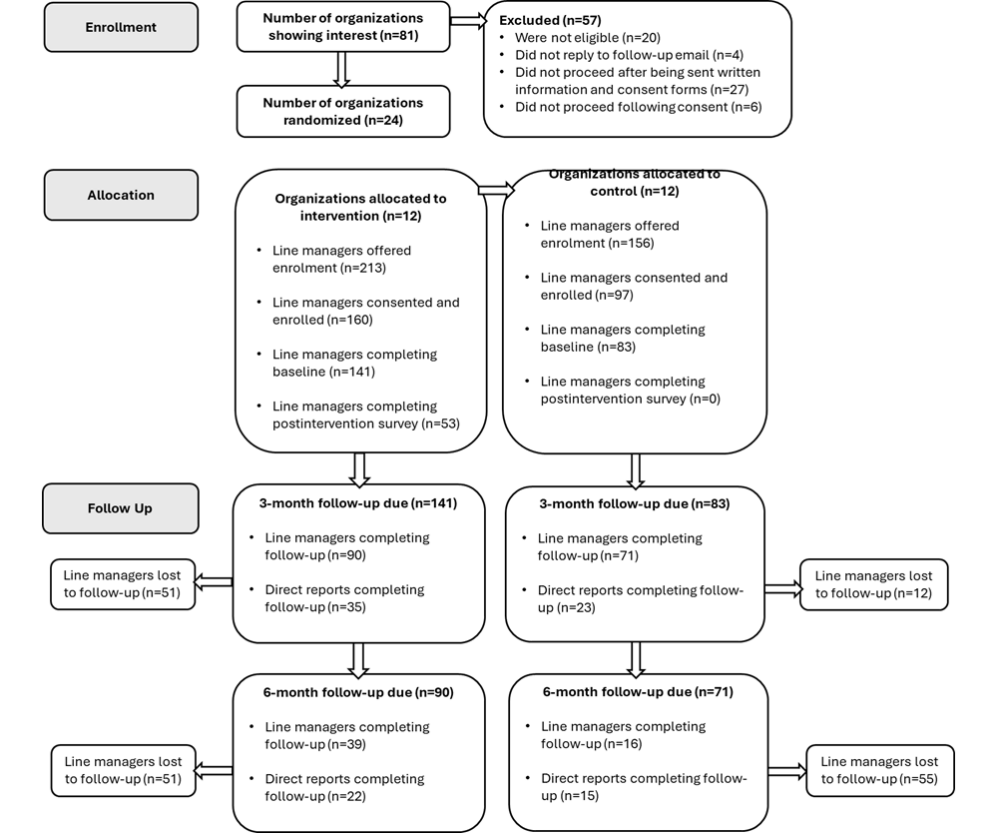
Participant flow diagram.

Organizations were recruited into the study via study websites, newsletters, and social media posts, and formal consent from organizational representatives was obtained. Line managers within each of the consenting organizations were contacted directly by the research team to invite them to participate in the study. Inclusion criteria for line managers were that they must be aged ≥18 years, have responsibility as a manager or supervisor, and have access to a work computer or mobile phone and an email address. Exclusion criteria for line managers were being due to retire or be made redundant in the following 6 months or having completed training on mental health at work in the previous 6 months. As this was a pilot feasibility trial, we aimed to recruit a minimum of 8 clusters (randomized into 2 groups of 4) and 30 line managers per arm to inform the parameters for a possible future trial. We exceeded these targets, recruiting 24 organizations and enrolling 141 line managers in the intervention arm and 83 in the control arm. A detailed overview of participant flow is provided in Figure 2.

### Intervention

The intervention is reported in line with the Template for Intervention Description and Replication checklist [47] (Multimedia Appendix 2) and the published protocol [46]. MMW was developed using a collaborative participatory design process over a period of 6 months from February 2021 to July 2021 [48]. It is a self-guided, stand-alone, web-based training intervention that asks recipients to work through 5 training modules. Table 2 provides a summary of MMW module content and empirical grounding. The study by Blake et al [48] provides further details on the module development process, content, and empirical grounding.

**Table 2:** Overview of the Managing Minds at Work training modules and theoretical foundations.

Module number and title	Summary description	Theoretical basis or empirical grounding
Introduction	Introduces the concept of mental well-being at work, the business case for focusing on mental health, and the legal responsibilities of line managers	Legal and organizational frameworks and introductory grounding for subsequent modules
Module 1: “Looking After Your Own Mental Health”	Focuses on self-care strategies for line managers, such as movement, flexible working, gratitude, and self-compassion	Self-care theory, reflective practice, and stress management literature
Module 2: “Designing and Managing Work to Promote Mental Well-being”	Covers the HSE’s^a^ Management Standards (demands, control, support, relationships, role, and change) on how to address and manage these work stressors through job design and management practices	HSE [49] Management Standards for work-related stress, primary prevention, and job design theory
Module 3: “Management Competencies that Prevent Work-related Stress”	Focuses on 4 key managerial competencies: respectful and responsible behavior, communication and workload management, handling difficult situations, and managing individuals	Management competencies for preventing work-related stress [50-52] and leadership and stress prevention literature
Module 4: “Developing a Psychologically Safe Working Environment”	Explores psychological safety, trust, inclusion, positive team relations, and peer support using interactive activities	Psychological safety theory [53] and team climate and inclusion literature
Module 5: “Having Conversations About Mental Health at Work”	Teaches communication skills to initiate, maintain, and respond in conversations about mental health with staff	Supportive communication models, mental health conversation frameworks, supporting openness about mental health, and behavior change theory

^a^HSE: Health and Safety Executive.

The web application used to host the MMW digital training (Xerte software; The Xerte Project [54]) was fully automated and did not provide the user with any contact with a therapist or coach. The training was hosted using the Xerte Online Toolkits platform (version 3.10; Apereo Foundation; accessed February 2022), a free, open-source tool designed for interactive content creation. Each module included written content but also interactive (eg, quizzes to test their knowledge) and reflective (eg, case studies) exercises to support learning. We did not include film and audio recordings as part of the training package due to budgetary restrictions. Module content was shaped by stakeholder feedback [48] and was designed to take 20 to 30 minutes to complete. Users were encouraged to complete 1 module per week (estimated intervention completion time: 5 to 6 weeks) and in the specified chronological order. The participating organizations agreed to allow line managers to complete this training during their working hours.

Once the baseline survey was completed, line managers in the intervention arm were provided with a username and log-in details for the training materials by a member of the research team. Line managers were asked to complete an end-of-module feedback form. These forms provided a proxy record of module completion by users as the software used did not allow for this functionality, and their completion was voluntary. These forms included closed (*yes* or *no* and Likert scales) items, as well open-ended questions to explore participants’ views on the module’s relevance, utility, acceptability, and content clarity.

During the intervention completion stage, the research team sent email reminders to encourage and remind line managers to work through the modules. A reminder email was sent once every 2 weeks. A maximum of 2 reminder emails were sent to participants.

### Randomization

The onboarded organizations were randomly assigned to either the intervention (the MMW digital training) or 3-month waitlist condition using an online random number generator [55]. This allocation was stratified to ensure a spread of different organization sizes within the intervention and control arms. Each organization was allocated a number, and 50% of the numbers generated were assigned to the intervention group, with the remaining 50% assigned to the waitlist control group.

### Procedure for Data Collection

Participants provided electronic informed consent by actively selecting a checkbox on the web-based survey platform before proceeding to the baseline assessment. Follow-up meetings were conducted with key organizational contacts to address any questions and confirm participation. In all cases, formal consent was obtained from senior management, indicating organizational-level support for the study. As part of this onboarding procedure, organizations agreed to provide time and support for the line managers in completing the training but were not informed of which line managers chose to participate in the study.

Once consent was obtained by the employing organizations, they were asked to provide a list of line managers with contact details. Once this list was received, a member of the research team contacted the line managers directly to invite them to participate in the study. The onboarded line managers received a link to the line manager baseline survey, the participant information sheet, and the consent form via their preferred email address.

To maximize engagement with the MMW digital training intervention, line managers were given flexibility as to when they completed the baseline survey and started the training intervention. Therefore, opening and closing dates varied across organizations (opening date: October 13, 2021; closing date: March 31, 2022). Onboarded line managers supported the study’s recruitment of direct reports by sending them an email with information about the study and a link to the baseline survey. These communications were drafted by the research team. However, to ensure confidentiality, the line managers were not informed of which direct reports opted into the study. No incentives were offered to direct reports due to budgetary constraints. Nevertheless, line managers and participating organizations agreed to support the study by allowing both direct reports and line managers time during their working day to complete the study materials if they wished. All feedback mechanisms provided direct report findings aggregated across the study, not by organization.

Both line managers and their direct reports across experimental conditions were sent a follow-up survey 3 months (closed on June 22, 2022) and 6 months (closed on October 7, 2022) following the completion of their baseline measure using their preferred email address as indicated on their completed consent form. In the control arm, line managers were asked to complete the 3-month measurement before they were given access to the intervention. The completed surveys were linked using the participants’ assigned username (eg, managingminds1, managingminds2, and so on) for the web application. All log-in details for users were unique and were not connected to their name or work site but, rather, to their preferred email address as provided on the consent form. Most participants chose their work-connected email addresses. The study participants were informed about the security of the web-based application and how their provided data would be securely stored and managed. Information provided as part of the interactive exercises on the platform was not held or stored.

Following the completion of the 6-month survey, a member of the research team contacted the participating organizations to see whether they were willing to share their sickness absence data for direct reports associated with the line managers in the trial. Line managers in the intervention arm and organizational stakeholders in both arms were invited to take part in semistructured interviews to gather qualitative data on their experience of the intervention and its acceptability, usability, and utility. The interviews were conducted using Teams (Microsoft), recorded, and transcribed verbatim.

### Feasibility Outcomes

Table 1 shows the data sources and methods used to address each of the feasibility objectives (objectives 1.1, 1.2, 1.3, 2.1, and 2.4). In summary, end-of-study records were used to assess recruitment of organizations into the trial (objective 1.1), recruitment and retention rates for line managers (objective 1.2), and recruitment and retention rates for direct reports (objective 1.3). The acceptability, usability, and utility of the MMW web-based training intervention among line managers and stakeholders (objective 2.1) and barriers to and facilitators of implementation of the intervention (objective 2.4) were assessed through qualitative interviews and web-based feedback forms completed at the end of each of the 5 MMW modules.

### Primary and Secondary Outcomes

To assess objectives 2.2 and 2.3 (Table 1), we measured a series of outcomes for both line managers and direct reports to examine the potential effectiveness of MMW to inform the design of a full trial. Improvement in line manager outcomes (objective 2.2) was assessed by measuring their confidence to foster a healthy workplace, mental health knowledge and literacy at work, psychological well-being, and self-rated management competencies to prevent work-related stress. Improvement in direct report outcomes (objective 2.3) was assessed by measuring their psychological well-being, self-rated productivity, perceptions of their line managers’ management practices and behaviors, and sickness absence. Table 3 provides an overview of the study measures.

**Table 3:** Assessment schedule for line managers (LMs), direct reports (DRs), and organizational measures.

Group and measure		Baseline	3 mo after baseline	6 mo after baseline
**LMs**				
	Demographics	✓		
	Confidence to create a mentally healthy workplace [56]	✓	✓	✓
	Mental health knowledge [57]	✓	✓	✓
	Workplace mental health literacy [58]	✓	✓	✓
	Stress Management Competency Indicator Tool—manager version [50]	✓	✓	✓
	Psychological well-being (WEMWBS^a^) [59]	✓	✓	✓
**DRs**				
	Demographics	✓		
	Psychological well-being (WEMWBS) [59]	✓	✓	✓
	Stress Management Competency Indicator Tool—employee version [50]	✓	✓	✓
	Self-reported productivity rating	✓	✓	✓
**Organizational level**				
	Sickness absence data trends			✓

^a^WEMWBS: Warwick-Edinburgh Mental Well-Being Scale.

Confidence in managing mental health issues and promoting a mentally healthy workplace was measured using a previously published supervisor scale [60] later modified by Gayed et al [56]. This is a 6-item measure describing various workplace scenarios such as “creating a work environment that prevents and reduces stress within my team.” Line managers were asked to rate their confidence in dealing with each of these scenarios on a 5-point Likert scale ranging from *not at all* to *extremely confident*. This measure has a minimum score of 6 and a maximum score of 30, with higher scores indicating increased confidence in creating a mentally healthy workplace. This measure has demonstrated good psychometric properties previously and been validated against manager behavior [61]. The Cronbach α was 0.84 at baseline.

Mental health knowledge was measured using the Mental Health Knowledge Schedule [57], a 6-item questionnaire rated on a Likert scale from 1 to 6 (1=*strongly disagree*; 5=*strongly agree*; 6=*I don’t know*). In line with scale guidance, responses of *I don’t know* (6) were recoded to *neutral* (3). The sixth item was reverse coded before creating a composite score. Higher composite scores were suggestive of better mental health knowledge (scale range 6-36) The 6 key areas explored by the scale include help seeking, recognition, support, employment, treatment, and recovery. The reliability of this scale was weak in our line manager sample at baseline (Cronbach α=0.40).

The Mental Health Literacy Tool for the Workplace [58] is a 16-item measure using vignettes tailored for the workplace context completed by line managers. This measure consists of vignettes about various mental ill-health scenarios within the workplace, with parallel questions that explore each of the 4 dimensions of mental health literacy. Items are ranked on a 5-point Likert scale ranging from *very low* to *very high*. A higher composite score indicates higher levels of workplace mental health literacy (scale range 16-80). Previous research has found good psychometric properties for this scale [62]. Our baseline sample showed a good internal consistency for this measure (Cronbach α=0.90).

The Warwick-Edinburgh Mental Well-Being Scale is a 14-item measure that seeks to quantify psychological well-being [59]. This measure was completed by both line managers and their direct reports. It asks participants to rank their psychoemotive experience in the previous 2 weeks across a series of statements (eg, “I have been feeling useful”) using a 5-point Likert scale ranging from *none* to *all of the time*. Items are summed to obtain a total score (score range 14-70). Higher scores are indicative of better overall psychological well-being. Strong psychometric properties have been found for this scale using community-based samples (eg, Cronbach α=0.91 [59] and Cronbach α=0.93 [59]). At baseline, in both our line manager and direct report samples, strong internal consistency was observed (line manager Cronbach α=0.92; direct report Cronbach α=0.90).

Management competencies for work-related stress were measured among both line managers and their direct reports. The Stress Management Competency Indicator Tool (SMCIT [50-52]) aimed to explore the behavioral competencies of line managers surrounding the prevention and management of work-related stress. The SMCIT has 2 versions, one for line managers to complete to rate their own competencies and an employee (direct report) version. The items are mirrored in both variants, with phrasing altered to suit the target audience (eg, “When necessary, I stop additional work being taken on by my team” [manager version] and “My manager will, when necessary, stop additional work being passed on to me” [employee version]). The SMCIT is measured on a 5-point Likert scale ranging from 1 (*strongly disagree*) to 5 (*strongly agree*) and consists of 66 items. This measure explores 4 key competency areas: managing emotions and having integrity (17 items, scale range 17-85), managing and communicating existing and future work (22 items, scale range 11-110), managing the individual within the team (15 items, scale range 15-75), and reasoning and managing difficult situations (12 items, scale range 12-60). Items in each subscale are summed to create a composite score for each subcompetency, with higher scores indicating better management competencies. Previous research has found satisfactory internal consistency (Cronbach α>0.7) for both line managers [63] and employees [64]. All our Cronbach α values were >0.7 in both our line manager and direct report groups.

Direct reports were asked to complete a single item on their self-reported productivity: “To what degree do you agree with the following statement? In the past week, I have been working under my average productivity,” anchored using a 5-point Likert scale ranging from *not at all* to *completely*. Lower scores represent better self-rated productivity by direct reports.

### Analytical Methods

SPSS (version 28; IBM Corp) [65] was used to conduct statistical analysis with collected quantitative data to address objectives 2.2 and 2.3. The significance level was set at *P*<.05. Missing data were inspected before data analysis to ensure that they were missing completely at random and individual cases did not have an excess of 5% of missing data [66]. Where missing data were identified and observed to be missing completely and not >5% per case, an imputation method was applied.

Descriptive statistics were calculated for each outcome measure with 95% CIs. Mean values were calculated at the participant (for line manager and direct report data; grand mean) and organizational (for line manager data only; group mean) levels. As this was a pilot feasibility trial, we did not use multilevel modeling. However, the intraclass correlation coefficient (ICC(1)) was calculated to examine the variability between clusters (organizations) to inform a future trial on the influence of these clusters on observed treatment effects.

Although our original protocol specified descriptive statistics only [23], we extended our analysis to include exploratory inferential statistics to examine preliminary group differences. This decision was justified by the fact that we substantially exceeded our initial recruitment target, enrolling 24 organizations and >220 line managers supported by additional project funding. While inferential statistics are not typically used in feasibility studies, the increased sample size enhanced our ability to detect preliminary trends and estimate effect sizes to inform future power calculations [67]. The purpose of these analyses was not hypothesis testing but rather to explore potential signs of change between arms and inform the design of a future definitive trial. We acknowledge this as a deviation from the original statistical analysis plan and recommend that future feasibility studies prespecify such analytic flexibility where appropriate.

We quantified and tested change over time across our experiential conditions, creating a new study variable for each outcome measure (line manager and direct report). This new variable was calculated by subtracting the participants’ mean score at the 3-month follow-up from their baseline measure. We did not use the 6-month follow-up measure to compare between the intervention and control groups as our waitlist control group had gained access to MMW at this point.

A 2-tailed independent *t* test was used to test for significant group differences between the intervention and control arms. Bootstrapping (bias corrected and accelerated with 5000 iterations) was used to adjust for deviations from multivariate normality and to yield 95% CIs [68]. Both *P* values (<.05) and CIs (not crossing 0) were inspected to assess statistical significance. We calculated the effect size for each comparative analysis using the Cohen *d* (with 95% CIs [69]). Drawing on the interpretive guidelines by Cohen [69] to support interpretation, values of *d* ≥0.2 were categorized as a small effect, values of *d*>0.5 to *d*≤0.8 were categorized as a medium effect, and values of *d*>0.8 were categorized as a large effect [68].

The data provided through the end-of-module feedback forms were analyzed descriptively to summarize participants’ views on the module’s relevance, utility, acceptability, and content clarity. Thematic analysis [70] was used for the qualitative data derived from the interviews. The detailed analytical methods and results of the qualitative analysis for trial objective 2.4 will be written up and published separately.

## Results

Figure 2 shows the flow of participants through each stage of trial. Our results are presented in this section by trial objective.

### Trial Objective 1: Potential Uptake and Interest in Participating in the Study by Employers, Line Managers, and Direct Reports

#### Objective 1.1: Willingness of Employers to Register Interest and Participate in the Study

We successfully recruited beyond our a priori specified sample size for participating organizations (n=8), ultimately enrolling 24 organizations across sectors and sizes within 6 months, demonstrating strong interest and feasibility for larger-scale implementation. Recruitment was open from August 20, 2021, to January 27, 2022. In total, 81 organizations expressed an interest in taking part in the MMW trial. Of these 81 organizations, 30 (37%) completed the organizational consent form, and 24 (30%) proceeded to fully enroll in the feasibility trial. Of the 57 organizations that did not take part, 20 (35%) were ineligible, 4 (7%) did not reply to the follow-up email, 27 (47%) did not proceed after being sent written information and consent forms, and 6 (11%) did not proceed following consent.

#### Objective 1.2: Recruitment and Retention of Line Managers

The recruitment of line managers from within the consented and participating organizations took place over 9 months. A total of 369 line managers were emailed information offering them enrollment in the MMW study. Of these 369 line managers, 257 (69.6%) consented to and enrolled in the study, with 224 (87.2%; n=141, 62.9% in the intervention arm and n=83, 37.1% in the control arm) completing the baseline survey. Most line managers recruited for the study (145/224, 64.7%) were female, with an average age of 44.7 (SD 8.3) years. On average, line managers had 10.7 (SD 7.4) years of management experience, ranging from <1 to 30 years. Table 4 provides the baseline demographics by experimental condition.

At 3 months, 71.9% (161/224) of the line managers who completed the baseline survey completed the follow-up assessment (intervention arm: 90/161, 55.9%; waitlist control arm: 71/161, 44.1%). At 6 months, 24.6% (55/224) of those who completed the baseline survey completed the follow-up assessment (intervention arm: 39/55, 71%; waitlist control arm: 16/55, 29%). Our line manager retention rates surpassed our a priori specified criteria of 70% at 3 months but not the 50% retention rate at the 6-month follow-up.

**Table 4:** Baseline line manager demographics by experimental condition.

		Intervention arm (n=141)	Control (waitlist) arm (n=83)
Age (y), mean (SD; SE; 95% CI; range)		44.6 (7.6; 0.6; 43.3-45.8; 26-61)	44.4 (8.6; 0.9; 42.5-46.2; 30-69)^a^
**Gender, n (%)**			
	Women	90 (63.8)	55 (66.3)^b^
	Men	51 (36.2)	28 (33.7)^b^
	Nonbinary	0 (0)	0 (0)
Tenure managing people (y), mean (SD; SE; 95% CI; range)		10.0 (6.7; 0.6; 8.9-11.2; 0-30)	12.2 (8.4; 0.9; 10.4-14.0; 0-30)

^a^n=84.

^b^n=83.

#### Objective 1.3: Recruitment and Retention of Line Managers’ Direct Reports

The recruitment of direct reports took place over 7 months. Due to expected variations in the number of direct reports per line manager, we did not set a priori targets for the number of direct reports to be recruited. Furthermore, we are not aware of how many direct reports were approached by their line managers to participate and, therefore, cannot calculate the percentage recruited to the study. Table 5 provides an overview of the demographics of direct reports at baseline. A total of 112 direct reports completed the baseline survey (intervention arm: n=74, 66.1%; waitlist control arm: n=38, 33.9%). At baseline, 78.6% (88/112) of direct reports were female, with an average age of 41.4 (SD 10.29) years and a mean tenure of 7.8 (SD 7.9) years in the organization. At 3 months, 51.8% (58/112) of the direct reports who completed the baseline survey completed the follow-up assessment (intervention arm: 35/58, 60%; waitlist control arm: 23/58, 40%). At 6 months, 33% (37/112) of the direct reports who completed the baseline survey completed the follow-up assessment (intervention arm: 22/37, 59%; waitlist control arm: 15/37, 41%).

**Table 5:** Baseline direct report demographics by experimental condition.

		Intervention arm (n=74)	Control (waitlist) arm (n=38)
Age (y), mean (SD; SE; 95% CI)		42.7 (9.92; 1.2; 40.5-44.9)	38.95 (10.66; 1.63; 35.6-42.3)
**Gender, n (%)**			
	Female	58 (81.7)^a^	30 (78.9)
	Male	13 (18.3)^a^	7 (18.4)
	Prefer not to say	0 (0)	1 (2.6)
Tenure (y), mean (SD; SE; 95% CI)		8.95 (8.59; 1.0; 6.9-10.9)	5.67 (6; 1.0; 3.9-7.7)

^a^n=71.

### Trial Objective 2: The Perceived Suitability and Potential Effectiveness of the Line Manager Training

#### Objective 2.1: Acceptability, Usability, and Utility of the MMW Digital Training Intervention

Qualitative data from postintervention interviews with line managers and stakeholders and qualitative feedback provided by the line managers after each training module were used to assess the acceptability, usability, and utility of the MMW digital training intervention. A total of 20 interviews were conducted (n=16, 80% line managers; n=4, 20% organizational gatekeepers). Feedback forms were completed by >100 line managers after each module (104/224, 46.4%-169/224, 75.4% across the 5 modules), and descriptive data are presented in Table 6.

**Table 6:** Descriptive data from module feedback forms completed by line managers (LMs) in the intervention and waitlist control arms.

		Feedback on each module, n/N (%)				
		Module 1—“Self-Care for LMs” (n=169)	Module 2—“Designing Work to Prevent Stress” (n=145)	Module 3—“Management Competencies” (n=112)	Module 4—“Conversations on Mental Health” (n=112)	Module 5—“Psychological Safety at Work” (n=104)
**Was the module relevant to your managerial role?**						
	Yes	160/164 (97.6)	139/140 (99.3)	110/111 (99.1)	110/110 (100)	101/101 (100)
	No	4/164 (2.4)	1/140 (0.7)	1/111 (0.9)	0 (0)	0 (0)
**Did you learn anything that you did not know before?**						
	Yes	84/166 (50.6)	117/142 (82.4)	76/110 (69.1)	82/111 (73.9)	92/100 (92)
	No	82/166 (49.4)	25/142 (17.6)	34/110 (30.9)	29/111 (26.1)	8/100 (8)
**It was easy to find the time to complete the module.**						
	Strongly agree or agree	105/166 (63.3)	73/139 (52.5)	61/112 (54.5)	61/110 (55.5)	64/101 (63.4)
	Neither agree nor disagree	27/166 (16.3)	28/139 (20.1)	20/112 (17.9)	20/110 (18.2)	16/101 (15.8)
	Strongly disagree or disagree	34/166 (20.5)	38/139 (27.3)	31/112 (27.7)	29/110 (26.4)	21/101 (20.8)
**The module was an appropriate length for the information provided.**						
	Strongly agree or agree	159/165 (96.4)	136/139 (97.8)	107/112(95.5)	105/110 (95.5)	98/101 (97)
	Neither agree nor disagree	2/165 (1.2)	1/139 (0.7)	3/111 (2.7)	5/110 (4.5)	3/101 (3)
	Strongly disagree or disagree	4/165 (2.4)	2/139 (1.4)	2/111 (1.8)	0/0 (0)	0/0 (0)
**The web-based module was easy to navigate.**						
	Strongly agree or agree	158/164 (96.3)	126/139 (90.6)	109/112 (97.3)	103/111 (92.8)	97/100 (97)
	Neither agree nor disagree	2/164 (1.2)	6/139 (4.3)	2/112 (1.8)	7/111 (6.3)	2/100 (2)
	Strongly disagree or disagree	4/164 (2.4)	7/139 (5)	1/112 (0.9)	1/111 (0.9)	1/100 (1)
**I found some of the information presented difficult to understand.**						
	Strongly agree or agree	14/166 (8.4)	8/139 (5.8)	8/112 (7.1)	8/111 (7.2)	10/101 (9.9)
	Neither agree nor disagree	3/166 (1.8)	3/139 (2.2)	4/112 (3.6)	3/111 (2.7)	2/101 (2)
	Strongly disagree or disagree	149/166 (89.8)	128/139 (92.1)	100/112 (89.3)	100/111 (90.1)	89/101 (88.1)
**The examples provided were not relevant to my role as a manager.**						
	Strongly agree or agree	19/166 (11.4)	18/139 (12.9)	16/112 (14.3)	15/111 (13.5)	10/101 (9.9)
	Neither agree nor disagree	18/166 (10.8)	14/139 (10.1)	10/112 (8.9)	8/111 (7.2)	4/101 (4)
	Strongly disagree or disagree	129/166 (77.7)	107/139 (77)	86/112 (76.8)	88/111 (79.3)	87/101 (86.1)

In general, reactions were very positive and suggest that the intervention was perceived by both line managers and stakeholders as acceptable, usable, and useful. Line managers felt that the modules were relevant to their managerial roles (160/164, 97.6% to 101/101, 100% agreement across the 5 modules) and they learned new information from the content, especially from modules “psychological safety at work” (92/100, 92% agreement) and “designing work to prevent stress” (117/142, 82.4% agreement). Qualitative comments on the feedback forms supported these findings:

...this course has been excellent. I like to think that I am a good leader anyway, but this is giving me lots of ideas about how to be an even better one. Thank you!

There were high levels of agreement about the length of each module being appropriate (ranging from 107/112, 95.5% to 136/139, 97.9% agreement) and that the content was easy to navigate (ranging from 126/139, 90.6% to 109/112, 97.3% agreement). Interview participants liked the flexibility in the way in which the training was delivered and highlighted specific aspects that were particularly beneficial, for example, its ease of use and relevance to not only their team’s mental health but also their own self-care and mental health. Qualitative feedback collected from the module feedback forms echoed the sentiments expressed in the stakeholder interviews, confirming the acceptability, usability, and utility of the MMW digital training intervention. For example, one participant said the following:

I have learnt how to recognise signs of concerns for the mental wellbeing of my team and also friends and family outside of the workplace. It also allowed me to self-reflect on my own mental wellbeing. I now have many resources that I downloaded from this course and links to other websites for extended reading.

The convergence between these 2 data sources strengthens the validity of the findings, affirming the positive reception of the intervention by both the employing organizations and line managers.

#### Objective 2.2: Changes in Line Manager Outcomes

Table 7 provides a descriptive overview of observed means (at both the participant and cluster level), SDs, and calculated ICC(1) values for line manager outcomes across the time series. While these statistics are presented descriptively, the inclusion of the ICC(1) aligns with feasibility trial guidance to inform future trial design and sample size planning [23]. Given the clustered nature of our data (ie, line managers nested within organizations), the ICC(1) was calculated to assess the proportion of the variance attributable to clustering and the potential need for multilevel modeling in future trials. Across most outcome measures, the observed ICC(1) values ranged from 0% to 13%, with most falling below the commonly suggested thresholds of 5% to 12% for minimal clustering effects [69,71]. These findings suggest a limited need to adjust for clustering in subsequent analyses, with the possible exception of workplace mental health literacy, which exhibited a slightly higher ICC(1). These exploratory estimates provide a valuable foundation for determining whether multilevel modeling is warranted in a future definitive trial.

We observed statistically significant changes in the outcomes measured in the intervention group as compared to the control group from baseline to the 3-month follow-up across all our specified outcome measures (Table 8). In particular, we observed that line managers who completed MMW reported increased confidence to create a psychologically healthy workplace; better mental health knowledge, psychological well-being, and mental health literacy at work; and improved managerial competencies surrounding the prevention and management of work-related stress (being respectful and responsible, managing and communicating existing and future work, managing the individual within the team, and reasoning and managing difficult situations). The Cohen *d* was used to estimate the magnitude of this change over time as compared to the control group drawing from the interpretation benchmarks by Cohen [69] (small: *d*=0.2; medium: *d*=0.5; large: *d*=0.8). A moderate change in the intervention group as compared to the control group was observed in relation to their self-reported psychological well-being (*d*=0.5) and their management competency on being respectful and responsible (*d*=0.7). A large effect size was observed across the remaining outcome measures examined in this pilot feasibility trial, ranging from 0.85 (management competency and reasoning and managing difficult situations) to 3.1 (mental health knowledge). However, the reliability of the mental health knowledge scale was weak, and therefore, this observation should be interpreted with a certain amount of caution. No significant adverse events were observed or reported among line managers during the study.

**Table 7:** Mean (SD) values across trial outcomes at the participant and cluster levels and by intervention and control arm across the time series and intraclass correlation coefficients (ICC(1); between clusters) at baseline.

Variable (Cronbach α) and group				Baseline							3 mo							6 mo							ICC(1)	
				Mean (SD)			Participants, n (%)			Mean (SD)				Participants, n (%)			Mean (SD)				Participants, n (%)					
**Confidence to create a mentally healthy workplace (score of 6-30; 0.84)**																										0.07
	**Intervention**					141 (100)							86 (96)							39 (100)						
		Participant	20.7 (3.9)					24.1 (3.1)							24.7 (3.0)											
		Cluster	20.8 (1.6)					24.4 (1.3)							24.8 (1.0)											
	**Control**					83 (100)							71 (100)							16 (100)						
		Participant	20.4 (3.6)					20.7 (3.9)							24.9 (2.5)											
		Cluster	20.3 (1.5)					20.7 (2.3)							24.0 (2.1)											
**Mental health knowledge (score of 6-36; 0.51)**																										0.00
	**Intervention**					141 (100)							86 (96)							38 (97)						
		Participant	23.4 (2.9)					25.2 (2.9)							24.7 (2.2)											
		Cluster	23.4 (0.9)					25.1 (0.8)							24.8 (1.9)											
	**Control**					83 (100)							71 (100)							16 (100)						
		Participant	22.8 (2.9)					23.0 (3.0)							22.9 (4.6)											
		Cluster	22.8 (0.7)					23.0 (1.1)							22.4 (1.9)											
**Psychological well-being (score of 14-70; 0.92)**																										0.00
	**Intervention**					141 (100)							85 (94)							38 (97)						
		Participant	47.9 (8.1)					52.1 (7.1)							52.2 (8.8)											
		Cluster	48.0 (2.9)					52.0 (2.5)							52.2 (4.1)											
	**Control**					83 (100)							71 (100)							16 (100)						
		Participant	49.7 (7.8)					48.6 (8.6)							54.1 (8.3)											
		Cluster	49.5 (3.0)					48.8 (3.7)							54.2 (6.5)											
**Mental health literacy at work (score of 16-80; 0.94)**																										0.13
	**Intervention**					141 (100)							86 (96)							38 (97)						
		Participant	48.7 (9.1)					61.5 (7.2)							63.0 (7.6)											
		Cluster	48.79 (4.15)					61.37 (2.68)							63.00 (3.47)											
	**Control**					83 (100)							71 (100)							16 (100)						
		Participant	47.7 (8.8)					49.0 (8.9)							61.9 (5.3)											
		Cluster	47.6 (3.8)					49.1 (4.4)							61.9 (4.6)											
**SMCIT^a^: being respectful and responsible, managing emotions, and having integrity (score of 17-85; 0.68)**																										0.00
	**Intervention**					140 (99.3)							86 (96)							39 (100)						
		Participant	69.7 (5.3)					72.4 (5.6)							74.2 (4.7)											
		Cluster	69.7 (1.7)					72.4 (2.6)							74.3 (2.2)											
	**Control**					83 (100)							71 (100)							16 (100)						
		Participant	69.3 (5.1)					68.8 (5.6)							72.6 (7.8)											
		Cluster	69.3 (2.5)					68.8 (3.0)							71.0 (5.1)											
**SMCIT: managing and communicating existing and future work (score of 22** **–** **110; 0.87)**																										0.05
	**Intervention**					140 (99.3)							86 (96)							39 (100)						
		Participant	87.9 (7.1)					93.7 (8.2)							96.2 (7.6)											
		Cluster	87.9 (2.7)					93.6 (2.9)							96.2 (2.4)											
	**Control**					83 (100)							70 (99)							16 (100)						
		Participant	87.6 (8.4)					87.1 (8.2)							92.3 (9.9)											
		Cluster	79.0 (3.7)					87.2 (3.9)							92.3 (7.0)											
**SMCIT: managing the individual within the team (score of 15-75; 0.84)**																										0.0
	**Intervention**					141 (100)							85 (94)							37 (95)						
		Participant	61.6 (5.9)					65.2 (5.7)							65.8 (5.9)											
		Cluster	61.6 (2.0)					65.1 (2.8)							65.8 (2.1)											
	**Control**					83 (100)							71 (100)							16 (100)						
		Participant	61.9 (5.9)					60.5 (5.7)							63.3 (6.5)											
		Cluster	61.9 (2.1)					60.7 (2.9)							63.3 (5.1)											
**SMCIT: reasoning and managing difficult situations (score of 12-60; 0.79)**																										0.07
	**Intervention**					141 (100)							86 (96)							38 (97)						
		Participant	46.6 (4.9)					50.2 (5.1)							51.7 (4.7)											
		Cluster	46.6 (1.9)					50.1 (1.9)							51.7 (2.1)											
	**Control**					83 (100)							71 (100)							16 (100)						
		Participant	46.2 (5.4)					46.2 (5.4)							50.6 (4.5)											
		Cluster	46.1 (2.2)					46.3 (2.9)							50.6 (3.0)											

^a^SMCIT: Stress Management Competency Indicator Tool [60].

**Table 8:** Results of independent t tests comparing observed changes over time (3-month follow-up – baseline) in trial outcome variables for line managers.

Outcome	Intervention group			Control group			*t* test (*df*)		*P* value		Mean difference (95% CI BCa^a^)		Cohen *d* (95% CI BCa)
	Change, mean (SD)	Participants, n (%)	Change, mean (SD)		Participants, n (%)								
Confidence to create a psychologically healthy workplace	3.8 (3.2)	86 (96)	0.6 (3.2)		71 (100)	6.4 (155)		<.001		3.3 (2.2-4.3)		1.0 (0.7-1.4)	
Mental health knowledge	1.9 (3.0)	85 (94)	0.2 (2.9)		71 (100)	19.6 (154)		<.001		1.7 (0.8-2.6)		3.1 (2.7-3.6)	
Psychological well-being	3.6 (8.3)	84 (93)	−0.7 (7.7)		71 (100)	3.3 (153)		.003		4.30 (1.9-6.8)		0.53 (0.2-0.9)	
Mental health literacy at work	11.8 (8.9)	84 (93)	0.8 (6.2)		69 (97)	9.0 (147.5)^b^		<.001^b^		11.1 (8.6-13.4)		1.4 (1.1-1.8)	
SMCIT^c^—being respectful and responsible	2.9 (5.5)	84 (93)	−0.4 (3.9)		70 (99)	4.4 (149.1)^b^		<.001		3.3 (1.9-4.9)		0.7 (0.4-1.0)	
SMCIT—managing and communicating existing and future work	5.5 (6.9)	86 (96)	−0.6 (5.8)		68 (96)	5.8 (152)		<.001		6.1 (4.1-8.2)		0.95 (0.6-1.3)	
SMCIT—managing the individual within the team	3.1 (5.1)	84 (93)	−1.4 (4.25)		71 (100)	5.9 (153)		<.001		4.46 (3.0-6.0)		0.9 (0.6-1.3)	
SMCIT—reasoning and managing difficult situations	3.5 (4.4)	86 (96)	−0.3 (4.7)		71 (100)	5.3 (155)		<.001		3.8 (2.4-5.3)		0.85 (0.2-1.2)	

^a^BCa: bias corrected and accelerated.

^b^Significant Levene test; unequal variances reported.

^c^SMCIT: Stress Management Competency Indicator Tool.

#### Objective 2.3: Changes in Direct Report Outcomes

Table 9 provides a descriptive overview of the mean scores observed for direct reports within the intervention and control arms over the time-series data collection.

Due to the research design, we were not able to delineate which direct reports were derived from which line managers or participating organizations. Therefore, it is not possible to provide a mean at the cluster level. We did not observe a statistically significant change in the psychological well-being of direct reports over time in the intervention group as compared to the control group (Table 10).

However, the direct reports of managers who received the intervention did show overall yet nonsignificant improvements in their psychological well-being and in their perceptions of their line managers’ competencies in managing and communicating existing and future work, managing the individual within the team, and managing difficult situations. We did observe a statistically significant change in self-rated productivity; however, this was not in the anticipated direction, with improved self-rated productivity in the control group rather than the intervention group over time. Across the 4 management competencies, as observed and reported by direct reports, we did not observe a statistically significant change over time in comparison to the 2 experimental conditions.

Following the completion of the intervention, we contacted the participating organizations to see whether we could obtain organizational-level sickness absence records. Unfortunately, we were not able to secure access to these data, which calls into question the feasibility of accessing this type of data in this way in a future trial and identifies the need to have clearer data sharing protocols set up in advance. No significant adverse events were observed or reported among direct reports during the study.

**Table 9:** Outcome means and SDs for the intervention and control group direct reports across the time series.

Group			Baseline				3 mo				6 mo		
			Mean (SD)		Participants, n (%)		Mean (SD)		Participants, n (%)		Mean (SD)		Participants, n (%)
**Self-reported productivity (score of 1-5; higher score indicates poorer productivity)**													
	Intervention	1.8 (1.0)		74 (100)		1.6 (1.0)		35 (100)		1.8 (0.9)		22 (100)	
	Control	1.7 (1.0)		38 (100)		2.3 (1.3)		23 (100)		2.1 (1.3)		14 (93)	
**Psychological well-being (score of 14-70; lower score indicates poorer well-being)**													
	Intervention	46.1 (7.9)		74 (100)		47.3 (9.0)		35 (100)		47.3 (8.5)		22(100)	
	Control	47.0 (7.3)		38 (100)		47.1 (6.7)		23 (100)		41.5 (7.6)		14 (93)	
**SMCIT^a^—being respectful and responsible**													
	Intervention	28.3 (2.5)		74 (100)		28.2 (2.8)		35 (100)		28.5 (2.7)		22 (100)	
	Control	29.2 (2.4)		38 (100)		29.3 (3.0)		23 (100)		28.4 (2.5)		14 (93)	
**SMCIT—managing and communicating existing and future work**													
	Intervention	33.0 (4.1)		74 (100)		33.4 (3.8)		35 (100)		33.2 (4.5)		22 (100)	
	Control	32.8 (4.9)		38 (100)		31.2 (5.9)		23 (100)		31.3 (7.3)		14 (93)	
**SMCIT—managing the individual within the team**													
	Intervention	13.0 (1.5)		74 (100)		12.9 (1.8)		35 (100)		12.9 (2.1)		22 (100)	
	Control	12.4 (2.3)		38 (100)		11.8 (2.9)		23 (100)		11.9 (2.2)		14 (93)	
**SMCIT—reasoning and managing difficult situations**													
	Intervention	33.7 (5.1)		74 (100)		44.0 (5.5)		35 (100)		35.4 (5.8)		22 (100)	
	Control	33.4 (5.5)		38 (100)		32.6 (6.1)		23 (100)		33.6 (6.7)		14 (93)	

^a^SMCIT: Stress Management Competency Indicator Tool.

**Table 10:** Results of independent t tests comparing observed changes over time (3-month follow-up – baseline) across trial outcome variables for direct reports.

Outcome	Intervention group			Control group			*t* test (*df*)		*P* value		Mean difference (95% CI BCa^a^)		Cohen *d* (95% CI BCa)
	Change, mean (SD)	Participants, n (%)	Change, mean (SD)		Participants, n (%)								
Self-rated productivity	−0.2 (1.3)	35 (100)	0.5 (1.4)		23 (100)	−2.2 (65)		.02		−0.7 (−1.4 to −0.1)		−0.6 (−1.1 to −0.1)	
Psychological well-being	1.1 (6.8)	35 (100)	1.0 (5.5)		23 (100)	0.1 (65)		.95		0.1 (−2.9 to 3.2)		0.01 (−0.5 to 0.5)	
SMCIT^b^—being respectful and responsible	−0.1 (3.1)	35 (100)	0.1 (3.3)		23 (100)	0.0 (65)		.49		0.0 (−1.4 to 1.6)		0.1 (−0.5 to 0.5)	
SMCIT—managing and communicating existing and future work	0.2 (3.4)	35 (100)	−0.5 (2.7)		23 (100)	1.0 (65)		.16		0.8 (−0.7 to 2.1)		0.3 (−0.3 to 0.8)	
SMCIT—managing the individual within the team	−0.1 (1.5)	35 (100)	−0.2 (1.6)		23 (100)	0.1 (65)		.45		0.1 (−0.7 to 0.9)		0.0 (−0.5 to 0.5)	
SMCIT—reasoning and managing difficult situations	0.4 (4.0)	35 (100)	0.2 (4.8)		23 (100)	0.2 (65)		.43		0.2 (−2.1 to 2.5)		0.0 (−0.5 to 0.5)	

^a^BCa: bias corrected and accelerated.

^b^SMCIT: Stress Management Competency Indicator Tool.

#### Objective 2.4: Barriers to and Facilitators of Implementation

Qualitative methods were used to explore the barriers to and facilitators of intervention implementation and effectiveness to inform the delivery of the intervention for a full trial. Data showed that a commonly perceived barrier to intervention implementation was time constraints and perceived workload. Module feedback forms (Table 6) highlighted that between a fifth and a quarter of line managers disagreed with the statement that it was easy to find time to complete the modules (ranging from 34/166, 20.5% to 31/112, 27.7% across the 5 modules).

Qualitative comments on the feedback forms also showed that line managers were struggling to fit the training into their workday:

Finding time to do them difficult, so its late and I’m tired.

In interviews, some line managers also described that their completion of the training was hampered by a lack of ability to commit time to engage with MMW due to their existing workload. Although a suggested weekly schedule was provided, workload demands meant that, for many participants, this was not followed, which may have lessened their learning:

The biggest challenge was finding the time to do it...I actually ended up squashing them all together at the end, which is inevitable. And actually I don’t think that’s as good for learning as it would have been if I spent done a little bit each week.

The main facilitators to completing and accessing the MMW modules focused on the flexibility offered by the self-led learning process and the support from senior managers. Interview participants described the benefits of the structure of the 5 modules, which could be completed at a convenient time but with a suggested schedule of 1 module per week. This gave them the flexibility to complete the modules at a time of their choice:

It absolutely does work in terms of it being self-led so that you know got quite a lot of time to complete that training and they can stretch it around their other commitments.

Encouragement and support from senior managers within the organization also facilitated completion of the training and were key to overcoming some of the barriers related to time constraints. However, stakeholders also said that integrating the training into their other existing activities and adding on other activities led by the organization would have allowed for even more encouragement and reflection:

[It] needed more integration, but that is any learning intervention that you take...I probably should have said use module 4 as a kick start and then probably taken that into some practical role plays. I could easily have run. Use that as the base. Take that forward to try and get confidence up in the conversations.

### Trial Objective 3: Feasibility of the Data Collection Methods

The Mental Health Knowledge Schedule [57] demonstrated poor levels of reliability within our pilot feasibility trial. Therefore, we conclude that this measure should not be used in a full trial context. The remainder of our measures used to quantify primary and secondary outcomes demonstrated satisfactory psychometric properties. Collecting sickness absence data from the organizations was particularly challenging, and therefore, its utility as a data collection method is limited. Thus, alternative data collection methods may be required to quantify changes in sickness absence behaviors in a future trial. This may include using self-report data from the perspective of direct reports. We included a sufficient number of organizations from a clustering perspective; however, the number of line managers within each cluster varied considerably. Therefore, to statistically account for any clustering effects, a full trial will need to consider and achieve both quantity in terms of clusters and variability regarding individuals within each cluster that are retained over the time series. Direct reports were challenging to recruit and retain in the study, so alternative methods of recruitment will be considered in future trials.

## Discussion

This feasibility trial aimed to pilot the MMW digital training intervention and assess its acceptability and potential for improving line manager and direct report outcomes, as well as the feasibility for a future definitive trial. The objectives were to assess (1) the potential for uptake in small, medium, and large companies; (2) the perceived suitability and potential effectiveness of the line manager training; and (3) the feasibility of the data collection methods for primary and secondary outcome measures.

### Principal Findings

In relation to objective 1, recruitment of organizations into the trial was very successful, indicating a demand for this type of intervention. The recruitment and retention of line managers was also positive and surpassed the prespecified criteria at 3 months. However, the recruitment and, in turn, retention of direct reports was considerably more challenging, highlighting the need to review and amend both our recruitment methods and retention efforts among direct reports in a future trial.

In relation to objective 2, feedback through end-of-module forms and interviews revealed that the intervention was deemed highly acceptable, usable, and useful by organizations and line managers. The MMW intervention also demonstrated potential for improving line manager outcomes, with significant changes observed in the intervention group as compared to the control group of line managers across all our outcome measures. Line managers who completed the MMW digital training intervention reported increased mental health knowledge, mental health literacy at work, and confidence to foster a psychologically healthy workplace, as well as reporting increases in their own psychological well-being and management competencies to prevent work-related stress. Furthermore, the magnitude of these changes was moderate to large in nature. These potential improvements reflect enhanced job resources and management competencies, which, according to the JD-R model [22], are key factors in buffering job demands and preventing stress-related outcomes (such as depression and anxiety). These findings highlight the potential impact of this digital training intervention on line managers directly. However, the indirect impact of this digital intervention on direct reports was not observed. This may be a result of experienced challenges in both recruitment and retention but may also be a limitation of a shorter follow-up period of 3 months. It is also possible, in line with the intervention conceptual framework, that distal outcomes among direct reports may require more time to manifest because of upstream managerial behavior change.

Qualitative data showed that perceived barriers to engagement with the intervention were time constraints and perceived workload. Line managers proposed that uptake in participants was hampered by a lack of ability to commit time to engaging with MMW due to their existing workload. This highlights an inherent tension between job demands and resource availability, reinforcing the importance of supportive organizational structures and managerial autonomy, which are central tenets of the JD-R model. We will build on the findings of this aspect of the study, and phase 2 will explore how the intervention could be implemented more efficiently alongside other organizational practices and policies and maximize the support of senior managers, who appear to play an important role in facilitating the completion of MMW.

This pilot feasibility trial provides preliminary evidence of the usability and acceptability of the MMW digital training for line managers, its potential to improve outcomes, and the feasibility of the research design and process. This study highlighted some key considerations to inform the future delivery and evaluation of MMW in a large-scale trial.

### Results in Context With Other Research

These promising results for the MMW digital training intervention offer significant value, particularly when contextualized within the broader landscape of workplace mental health and management practices. Line manager training has been identified as a key target for intervention [1,10,15], yet few robust evaluations of interventions have been conducted [27,32,35,36,38-40]. Therefore, this study contributes to the growing body of evidence that organizations and line managers are keen to engage with interventions to improve skills and competencies for preventing poor mental well-being at work and that change in these critical areas can occur. By targeting behavioral and competency development in line managers, the intervention aligns with a primary prevention approach, shifting focus from reactive to proactive strategies to reduce mental health risks.

The success in recruiting beyond the specified sample size is an encouraging indicator of employer willingness to engage with mental health interventions targeting line managers. The need for such interventions is emphasized by statistics showing that 1 in 6 workers experience mental health issues annually in the United Kingdom [3] and by policy standards and guidance [10,14] that highlight the importance of a comprehensive approach to workplace mental health interventions, specifically those that incorporate primary, secondary, and tertiary strategies. However, this success does contrast with other studies that have faced challenges in recruiting organizations for workplace interventions due to issues such as perceived burden of the study [72], further stressing the growing recognition of the importance of mental health in the workplace. Although the retention rates of line managers in our study surpassed the prespecified criteria at 3 months, they did not surpass the prespecified criteria at 6 months, indicating a common methodological challenge of maintaining engagement over time in longitudinal studies [73]. Despite our efforts to minimize attrition, the decline in retention rates suggests that other areas of methodological refinement are needed to maximize participant retention.

Research suggests that awareness of an intervention’s efficacy does not necessarily indicate that the participants will accept it. As such, it is important to capture the perceived acceptability of such interventions [74]. The favorable reception of the MMW digital training intervention regarding acceptability, usability, and utility underscores the need to involve end users in the development and refinement of health interventions. This finding also aligns with those of the growing body of research that supports digital mental health interventions in the workplace [40,75-77].

Line managers who received the MMW training exhibited improvements in several outcomes relative to those who did not receive the training, including confidence to create a psychologically healthy workplace, understanding of mental health issues, psychological well-being, mental health literacy at work, and managerial behavioral competencies. Studies exploring such interventions have obtained similar findings [27,78,79]. These outcomes reflect key job resources that, in line with the JD-R model, buffer strain and support well-being—reinforcing the program’s theory of change. Equipping managers with mental health knowledge and stress prevention skills through interventions such as MMW can improve workplace mental health overall.

The perceived barriers of time constraints and workload impacting the uptake of workplace interventions have often been observed across studies focusing on workplace wellness programs [20,80] and health promotion in general [81]. This is particularly significant in the context of mental health interventions as the need for such programs is often greatest in high-stress environments. Consequently, there is a need for organizational strategies that address these structural barriers to participant engagement. This reflects the JD-R model’s emphasis on organizational context and the role of environmental demands and supports in shaping outcomes. This aligns with other findings on the implementation of workplace mental well-being interventions that have identified the importance of senior management support, time allocation for training, and continuity of efforts to implement interventions [51,82].

### Strengths, Limitations, and Future Directions

A review of UK-based pilot and feasibility studies registered on ISRCTN from 2013 to 2020 found that the median target sample size was 33 per arm but 57% of the studies did not reach this sample size target [83]. In contrast, this study achieved sample sizes of 90 (intervention) and 71 (control) at the 3-month follow-up, where between-group comparisons were possible. These sample sizes also compare favorably with those of full cluster RCTs of similar interventions, which have frequently had smaller line manager samples at the first follow-up point (eg, n=62 and n=64 at 3 months [36], n=41 and n=78 at 4 months [32], n=25 and n=19 at 6 months [38], n=88 and n=54 at 8 weeks [34], and n=24 and n=13 at 12 weeks [35] in the intervention and control groups, respectively).

Our study was a pilot feasibility trial to enable us to test whether the components would work (recruitment, randomization, intervention acceptability, and follow-up) [84] but also to assess whether MMW has the potential to improve outcomes [71]. On the basis of the observed sample sizes and effect sizes, we can conclude with reasonable confidence that the intervention has promise in improving outcomes for line managers. However, to assess the effectiveness and cost-effectiveness of the intervention, a full, sufficiently powered trial would be needed informed by the findings of this pilot feasibility trial. One of the strengths of this study is the moderate to large effect sizes for changes in line manager knowledge, skills, and competencies.

Our strategy led to overrecruitment for our pilot feasibility study, yielding a larger dataset than anticipated. We acknowledge that this level of recruitment may blur the boundary between a feasibility study and a definitive trial. However, we remain cautious in our interpretation and do not claim effectiveness or impact on primary outcomes. Instead, we report these findings descriptively and emphasize their role in informing the design, implementation, and progression criteria for a future fully powered RCT. In line with this, we extended our originally planned descriptive analysis to include exploratory inferential statistics, which constitutes a deviation from our registered protocol. This decision was driven by the opportunity to extract more meaningful insights from a larger-than-expected sample, and we have transparently reported this deviation in the manuscript. This approach is aligned with recent guidance [23,71] that recognizes that feasibility studies may generate exploratory outcome data but such data should not be used to draw definitive conclusions about effectiveness. Therefore, we position our findings as hypothesis generating and implementation informing.

Although the direct reports of managers who received the intervention showed slight improvements in psychological well-being and perceptions of their managers’ competencies, these improvements were not of the same magnitude as those for line managers. Future research will need to test whether the line manager changes observed can be cascaded to direct report outcome improvements within the time frame of the study. Future research in a full trial will need to extend the follow-up timescales to allow for this, as well as develop a more effective recruitment and retention strategy for direct reports. In the context of this study, we recruited direct reports through their line managers disseminating study information to them directly. This recruitment approach may have acted as a barrier to direct report participation in the study due to potential concerns regarding their anonymity within the context of the study given that they would be reporting on their line managers’ behaviors and competencies. We will review and amend both our recruitment methods and retention efforts among direct reports in any future trial. A future trial may seek to recruit direct reports directly by a member of the research team either remotely or through workplace site visits.

This study did not use proactive efforts to retain direct reports in the study, only line managers. A future definitive full trial should consider retention efforts and activities directed at both line managers and their direct reports both independently and concurrently to support retention efforts. As this was a feasibility trial, we have identified a number of limitations to our original research design, which have been noted previously. These focus on the low recruitment of direct reports and the challenges in accessing organizational records for sickness absence. Additional limitations identified include the mental health knowledge scale showing poor levels of reliability, and we concluded that this measure should not be included in a full trial. Previous research has observed mixed findings in relation to the psychometric strength of this scale [57,85]. All other measures used to quantify primary and secondary outcomes demonstrated satisfactory psychometric properties. Collecting sickness absence data from organizations was particularly challenging, and therefore, its utility as a data collection method is limited.

Most line managers who opted into our study (145/224, 64.7%) were female and, due to the voluntary nature of the intervention, may have already been interested or invested in the importance of mental health at work. Therefore, a future trial should actively consider how to engage line managers identifying with other genders, as well as those less engaged or interested in the topic area of mental health at work. The self-selection of participants meant that participants were likely to be line managers who were interested in learning and motivated to learn about how they could develop their own skills and competencies in this area. The sample was limited to leaders and employees from UK-based organizations, which may constrain the generalizability of the findings. Future research should consider cross-cultural replications to explore how the intervention performs across different national, cultural, and organizational settings.

The web-based application would not allow us to track participants through the modules as they completed them due to budgetary constraints. Therefore, we are not able to report the proportion of modules completed by participants or the average completion time. Future work on this intervention will seek to address some of these limitations through developing a more sophisticated delivery platform that will enable tracking and feedback to participants on their completion progress throughout the MMW intervention. Moreover, to enhance engagement with the intervention, future studies could consider the use of behavioral nudges (eg, SMS text message reminders), interactive content (eg, quizzes and simulations), personalized messaging, incentive structures (eg, prize draws), and features that promote community building among participants. These elements may help increase uptake and sustained use of the intervention materials.

### Conclusions

This pilot feasibility cluster RCT demonstrated that the MMW digital training intervention had good acceptability, usability, and utility and that organizations and line managers could be recruited to and retained within a trial. Furthermore, MMW demonstrated that it has the potential to improve line managers’ confidence and competencies to prevent poor mental health among those they manage. A future full trial will assess the impact of these improvements over a longer period and how they may influence direct reports.

## Appendix

Multimedia Appendix 1 CONSORT-eHEALTH checklist (V 1.6.1).

Multimedia Appendix 2 Template for Intervention Description and Replication checklist.

## Abbreviations

CONSORT-EHEALTH: Consolidated Standards of Reporting Trials of Electronic and Mobile Health Applications and Online Telehealth
ICC(1): intraclass correlation coefficient
JD-R: job demands–resources
MMW: Managing Minds at Work
RCT: randomized controlled trial
SMCIT: Stress Management Competency Indicator Tool

## Data Availability

The data sets generated and analyzed during this study are available from the corresponding author upon reasonable request.
