# Supplementary material for: Web-Based Interactive Training for Managers (Managing Minds at Work) to Promote Mental Health at Work: Pilot Feasibility Cluster Randomized Controlled Trial
Source: JMIR Ment Health. 2025 Sep 2;12:e76373. doi: 10.2196/76373 (PMC12441645; doi:10.2196/76373)
Supplement: Multimedia Appendix 2 [file mental_v12i1e76373_app2.pdf]

TIDieR Checklist for *Managing Minds at Work*

Item	Description
**1. Brief Name**	Managing Minds at Work (MMW) digital training intervention.
**2. Why (Rationale, Theory, Goal)**	To equip line managers with skills, knowledge, and competencies for the primary prevention of poor mental health in the workplace. Developed in response to evidence gaps in prevention-oriented mental health training for line managers.
**3. What: Materials**	Web-based modules developed using Xerte Online Toolkits (Version 3.10, accessed February 2022). Materials included written content, quizzes, reflection exercises, and case studies. No film or audio materials included due to budget constraints.
**4. What: Procedures**	Intervention comprised five online self-guided modules: 1) Looking after your own mental health; 2) Designing and managing work to promote mental well-being; 3) Management competencies to prevent work-related stress; 4) Developing a psychologically safe work environment; 5) Having conversations about mental health at work. Participants received reminder emails during the training phase (maximum two reminders).
**5. Who provided**	The intervention was fully automated. No live facilitators, therapists, or coaches were involved. Research team provided initial login details and technical support if required.
**6. How**	Delivered via a secure web-based platform (Xerte), accessed individually using assigned usernames and passwords. Training was self-paced.
**7. Where**	Online, accessible via desktop computers, laptops, tablets, and mobile phones. Participants were permitted to complete the modules during working hours, per employer agreement.
**8. When and how much**	Participants were advised to complete one module per week over five weeks. Each module was designed to take approximately 20–30 minutes to complete.
**9. Tailoring**	No formal tailoring to individual participants. Standardized modules were provided to all users. Flexibility allowed participants to choose when they completed the modules within the suggested timeframe.
**10. Modifications**	No modifications to the intervention content during the trial. The training platform was "frozen" during the trial period to ensure consistency.

Item	Description
**11. How well: Planned adherence/fidelity assessment**	Completion of end-of-module feedback forms used as a proxy measure of module completion. No backend tracking of module access due to software limitations.
**12. How well: Actual adherence/fidelity**	Feedback forms indicated strong engagement with the modules. High rates of completion for feedback forms across all five modules were reported (n=104–169 forms completed).
